# Clinical features and course of *Angiostrongylus cantonensis* eosinophilic meningitis in patients receiving supportive therapy

**DOI:** 10.1016/j.fawpar.2020.e00095

**Published:** 2020-09-16

**Authors:** Kanlayanee Sawanyawisuth, Kittisak Sawanyawisuth, Vichai Senthong, Panita Limpawattana, Pewpan M. Intapan, Wanchai Maleewong, Thidarat Prasongdee, Sureeporn Naonongwai, Somsak Tiamkao, Verajit Chotmongkol, Sittichai Khamsai

**Affiliations:** a Department of Biochemistry, Faculty of Medicine, Khon Kaen University, Khon Kaen, Thailand; b Department of Medicine, Faculty of Medicine, Khon Kaen University, Khon Kaen, Thailand; c Department of Parasitology, Faculty of Medicine, Khon Kaen University, Khon Kaen, Thailand

**Keywords:** Eosinophilic meningitis, *Angiostrongylus cantonensis*, Predictors, Headache

## Abstract

Acute severe headache is the main presentation of eosinophilic meningitis (EOM) caused by *Angiostrongylus cantonensis*. Oral corticosteroid treatment is effective in reduction of duration of headache but may be contraindicated in particular patients. This study investigated clinical features and clinical course of eosinophilic meningitis caused by *A*. *cantonensis* if left untreated. Additionally, factors associated with duration of headache were evaluated. We conducted a retrospective study between 1997 and 2019 at a university hospital in Thailand. The inclusion criteria were adult patients who were diagnosed with EOM, had a positive serological test for *A*. *cantonensis*, received only supportive treatment, and had the complete clinical course documented. Factors associated with duration of headache were executed by multivariate linear regression analysis. A total of 54 patients were used in the final analysis. Of those, 39 patients (79.2%) were male and the mean ± SD age of all patients was 33.7 ± 12.2. The mean ± SD duration of headache was 16.0 ± 12.4 days with the longest duration of 49 days. The only factor associated with duration of headache was gender (*p* = 0.036). The male gender had a coefficient of −8.4 (95% CI: −16.2, −0.6). The median duration of headache in male and female patients was 11 and 20 days, respectively. In conclusion, *A*. *cantonensis* eosinophilic meningitis can cause long lasting headache, and gender may be associated with duration of headache.

## 1 Introduction

*Angiostrongylus cantonensis* is the main cause of eosinophilic meningitis, an emerging infectious disease worldwide (Ramirez-Avila et al., 2009). Humans become infected by consumption of uncooked freshwater snails, slugs, or contaminated foods such as juice or salad (Slom et al., 2002; Lv et al., 2017; Khamsai et al., 2020). The three main forms of *A*. *cantonensis* infection include eosinophilic meningitis, eosinophilic meningoencephalitis, and ocular angiostrongyliasis (Sawanyawisuth and Sawanyawisuth, 2008). Undiagnosed or missed diagnosed cases of eosinophilic meningitis may have risk for encephalitis development particularly in the elderly (Sawanyawisuth et al., 2009). Ocular manifestations are rare but can cause permanent visual loss (Sawanyawisuth et al., 2007).

Acute headache is the most common presentation of *A*. *cantonensis* infection. Corticosteroid is the cornerstone treatment in eosinophilic meningitis as evidenced by a randomized-controlled trial. A 2-week course of oral prednisolone significantly reduced duration of headache, use of analgesics and the number of lumbar punctures compared with placebo (Chotmongkol et al., 2000). However, corticosteroid may be contraindicated in some patients such as those with diabetes or immunocompromised conditions. Furthermore, the history of natural *A*. *cantonensis* infection has never been completely investigated. Here, we studied clinical features and clinical course of untreated eosinophilic meningitis caused by *A*. *cantonensis*. Additionally, factors associated with duration of headache in this study population were evaluated.

## 2 Materials and methods

This study was a retrospective study conducted at Khon Kaen University Hospital, Khon Kaen, Thailand. The study period was between 1997 and 2019. The inclusion criteria were adult patients who were diagnosed with eosinophilic meningitis and had positive serological test for *A*. *cantonensis* (Sawanyawisuth et al., 2011), received only supportive treatment, and had the complete recovery of headache documented. The serological test used for the detection of *A*. *cantonensis* was a 29-kDa antigenic band by immunoblotting. Sensitivity and specificity for the positive antigenic band were 55.6% and 99.4%, respectively (Maleewong et al., 2001). The supportive treatment for eosinophilic meningitis was only acetaminophen and/or lumbar puncture. Lumbar puncture was performed as needed to relieve headache. Corticosteroids or anthelminthics such as albendazole were not administered. For the purposes of this study, these patients were considered as having no treatment and may represent the natural progression of eosinophilic meningitis caused by *A*. *cantonensis*.

Baseline clinical factors included age, gender, incubation period, visual analogue scale (VAS) of headache, duration of headache, nausea/vomiting, paresthesia, and physical signs such as fever, neck stiffness, cranial nerve palsies, or papilledema. The VAS of headache is a self-reported severity of headache with a range of 0–10: 0 for no headache and 10 for the worst headache. Laboratory results including complete blood count and cerebrospinal fluid (CSF) analyses were evaluated. These laboratory tests were results at the time of diagnosis of *A*. *cantonensis* eosinophilic meningitis.

Numerical factors with normal distribution were presented as mean ± SD, while not normally distributed numerical factors were reported as median (range). Duration of headache in days of each patient was the primary outcome. Baseline factors were analyzed by univariate and multivariate linear regression to find the association with duration of headache. Factors with a *p* value of less than 0.20 by the univariate linear regression were included in the multivariate linear regression. A p value less than 0.05 was considered significant. Analytical results were presented as coefficient (95% confidence intervals; CI). All data analyses were performed with STATA software (College station, Texas, USA).

## 3 Results

Review of medical records revealed 55 patients presenting with eosinophilic meningitis, none of whom received supportive treatment; one of these patients was excluded due to incomplete clinical data. A total of 54 patients were included in the analysis. Of those, 39 patients (79.2%) were male and the mean ± SD age of all patients was 33.7 ± 12.2. All patients presented with acute severe headache (Table 1). The median duration of headache was 7 days (range 1–30) with the mean VAS of 9.2 out of 10. Only four patients (7.4%) had fever, while 27 patients (50%) had neck stiffness. Regarding laboratory results, the mean percentage of peripheral eosinophils was 19.5 ± 12.5%. The highest CSF white blood cell and protein were 2390 cells/mm^3^ and 470 mg/dL. The lowest CSF glucose was 20 mg/dL (Table 2).

**Table 1:** Clinical features of patients with eosinophilic meningitis caused by *Angiostrongylus cantonensis* who received only supportive treatment (*n* = 54) according to gender.

Clinical factors	All patients (n = 54)	Female group (*n* = 15)	Male group (*n* = 39)	p value
Age, year	33.7 ± 12.2	36 (16–56)	31 (16–63)	0.469
Incubation period, days	26.3 ± 18.9	30 (3–60)	21 (1–90)	0.251
Duration of headache, days	7 (1−30)	7 (3−21)	7 (1–30)	0.261
^⁎^VAS of headache	9.2 ± 1.1	10 (7–10)	10 (6–10)	0.207
Nausea/vomiting, N	30 (55.6)	10 (66.7)	20 (51.3)	0.370
Paresthesia, N	8 (14.8)	1 (6.7)	7 (18.0)	0.419
Fever, N	4 (7.4)	0	4 (10.3)	0.567
Neck stiffness, N	27 (50.0)	9 (60.0)	18 (46.2)	0.544
Cranial nerve palsy, N	1 (1.9)	0	1 (2.6)	0.999
Papilledema, N	1 (1.9)	0	1 (2.6)	0.999

**Note**. Data presented as mean ± SD, median (range), or numbers (percentage); ^⁎^VAS: **v**isual analogue scale; *p* values were differences between gender.

**Table 2:** Laboratory results and outcomes of patients with eosinophilic meningitis caused by *Angiostrongylus cantonensis* who received only supportive treatment (n = 54) according to gender.

Clinical factors	All patients (n = 54)	Female group (n = 15)	Male group (n = 39)	*p* value
White blood cells, cell/mm^3^ (5000–10,000)	12,701.0 ± 3992.7	12,200 (5200-18,300)	13,600 (4700-24,000)	0.635
% Blood eosinophils (0–6)	19.5 ± 12.5	17 (2–47)	12 (2–55)	0.841
CSF opening pressure, mmH_2_O (50–250)	271.1 ± 140.5	300 (100–600)	250 (30–600)	0.451
CSF closed pressure, mmH_2_O	157.4 ± 57.4	165 (50–280)	150 (15–280)	0.989
CSF white blood cell, cell/mm^3^ (<5)	849.3 ± 499.7	960 (340–1950)	780 (85–2390)	0.202
CSF eosinophils, % (0)	45.1 ± 19.9	43 (12–84)	46 (15–81)	0.629
CSF protein, mg/dL (<40)	125.3 ± 77.3	97 (27–183)	116 (39–470)	0.179
CSF sugar, mg/dL	50.7 ± 20.7	49 (25–134)	46 (20–107)	0.192
CSF sugar/blood sugar, % (>50)	46.4 ± 15.4	46.7 (29.4–100)	43.9 (16.9–103.9)	0.107
Numbers of required LP, times	1.8 ± 4.7	1 (0–7)	0 (0–27)	0.493
Duration of headache, days	16.0 ± 12.4	20 (4–47)	11 (1–49)	0.040

**Note**. Data presented as mean ± SD, median (range) or numbers (percentage); CSF: cerebrospinal fluid; LP: lumbar puncture; p values were differences between gender; number in parenthesis of the first column indicated normal values.

The mean ± SD duration of headache after supportive treatment was 16.04 ± 12.37 days and range of 1–49 days. The only factor associated with duration of headache by multivariate linear regression was gender. Males had coefficient of −8.4 (95% CI: −16.2, −0.6; *p* value 0.036) indicating that headache lasted 8.37 days shorter compared to female patients. The median duration of headache in male and female patients were 11 and 20 days, respectively (Table 2) (*p* = 0.04, Wilcoxon rank sum). Other clinical factors between male and female patients were compared by descriptive statistics and did not show any significant differences (Table 1, Table 2).

## 4 Discussion

In comparison to studies that included corticosteroid treatment and albendazole or mebendazole, the clinical features of *A*. *cantonensis* eosinophilic meningitis patients who received only supportive treatment were similar to previous studies (Chotmongkol et al., 2004; Chotmongkol et al., 2006). The most important clinical presentation was acute headache without any other neurological symptoms. Some clinical signs may be a crucial clue such as paresthesia, cranial nerve palsy (6th or 7th), or papilledema. However, these signs were uncommon (1–15%) as shown in Table 1. Even though CSF eosinophils are diagnostic criteria, clinical signs of meningism are rare for *A*. *cantonensis* eosinophilic meningitis. Fever and neck stiffness can be found in 7.4% and 50% of patients. Fever, headache, and neck stiffness are suggestive for bacterial meningitis, but these signs were not common in *A*. *cantonensis* eosinophilic meningitis. Therefore, mis- or under-diagnosis of this disease may occur. Peripheral eosinophila with greater than 798 cells may be suggestive for *A*. *cantonensis* eosinophilic meningitis with sensitivity of 76.6% (Sawanyawisuth et al., 2010). A lumbar puncture should be performed if *A*. *cantonensis* eosinophilic meningitis is suspected. Performing the lumbar puncture can be a diagnostic tool and serve to relieve intracranial pressure (Sawanyawisuth and Sawanyawisuth, 2008).

Clinical clue is history of consuming uncooked freshwater snails, slugs, or salads preceding headache development. In northeastern Thailand, an endemic area, physicians should routinely consider this risk in patients with acute severe headache. However, outbreaks may occur even in non-endemic areas, as reported in the US and China (Slom et al., 2002; Zhou et al., 2009). History of travel to endemic areas and possible exposure to *A*. *cantonensis* larvae is also crucial (Ansdell and Wattanagoon, 2018). Neglected headaches as a symptom of angiostrongyliasis can lead to the development of a more severe form of the disease. The adjusted odds ratio for encephalitis was 1.26 times per one day duration of headache (Sawanyawisuth et al., 2009). The issue with encephalitis development is crucial due to the high morbidity and mortality rate (Chotmongkol and Sawanyawisuth, 2002). Up to 80% of patients who developed encephalitis died (Chotmongkol and Sawanyawisuth, 2002).

This study also showed the natural history of headache character in eosinophilic meningitis caused by *A*. *cantonensis* who received only supportive treatment. If the patients received supportive treatment, the duration of headache after treatment was varied. The patients may improve in a single day or the headache may persist for up to 49 days. The mean duration of headache was 16 days. It should be noted that the severity of headache was assessed as most severe at the beginning with the visual analogue scale of almost 10 (9.21). A previous study also showed that at least 60% of 484 patients with eosinophilic meningitis still had headache after a 2-week course of analgesics (Punyagupta et al., 1975). The headache may improve over time, but slowly. However, the severe headache may disrupt the patient's daily life. Corticosteroids are beneficial and recommended to reduce the severity of headache in this disease (Chotmongkol et al., 2000; Ansdell and Wattanagoon, 2018).

The results of this study also indicate that female gender was significantly associated with longer duration of headache. This finding was similar to previous reports on the effect of gender and pain severity. Female patients may have less pain tolerance than males; a lower pain threshold was shown in female patients compared to male patients in 29 of 34 measurements (Ostrom et al., 2017). Further studies are required to confirm different durations of headache between males and females. However, these results may be used in clinical practice. Physicians may consider supportive treatment as in this study with male patients who had mild headache and contraindications for steroid therapy. The median duration of headache in these male patients may be 11 days - 6 days more than corticosteroid treatment. The average duration of headache in a recent prednisolone treatment study was 5 days (Chotmongkol et al., 2000).

In conclusion, acute severe headache can be long-lasting in untreated *A*. *cantonensis* eosinophilic meningitis. Gender may be associated with the duration of headache.

## Declaration of competing interest

The authors declare that they have no known competing financial interests or personal relationships that could have appeared to influence the work reported in this paper.
